# Key performance indicators for pre hospital emergency Anaesthesia - a suggested approach for implementation

**DOI:** 10.1186/s13049-019-0610-x

**Published:** 2019-04-11

**Authors:** James Raitt, James Hudgell, Henry Knott, Syed Masud

**Affiliations:** 1 Emergency Medicine and Pre Hospital Emergency Medicine, Health Education England Thames Valley, Thames Valley Air Ambulance, RAF Benson, Oxford, OX10 6AA UK; 2 Anaesthesia and Pre Hospital Emergency Medicine, Defence Deanery, Lichfield, UK; 3 Emergency Medicine, Health Education England Thames Valley, Oxford University Hospitals, Lichfield, UK; 4 Emergency Medicine and Pre Hospital Emergency Medicine, Trauma and Pre Hospital Care, Oxford University, Lichfield, UK

**Keywords:** Pre hospital, Emergency Anaesthesia, Key performance indicators

## Abstract

**Background:**

Pre-hospital Emergency Anaesthesia (PHEA) is regarded as one of the highest risk interventions that pre-hospital providers perform. AAGBI guidance from 2017 suggests the use of Key Performance Indicators (KPIs) to audit PHEA quality. The aim of this study was to develop KPIs for use in our service and evaluate their impact.

**Methods:**

Using the AAGBI 2017 document as a guide we developed a list of ten auditable domains. Data for each case was extracted from the Electronic Patient Record (EPR) and a score assigned to each of the domains; one if the domain is achieved and zero if the domain is not achieved or if data is missing, giving a total score out of ten. This analysis is then presented as a colour-coded matrix alongside the score. Data were analysed monthly at our case review and governance meeting. The process was refined during the year and after 12 months a formal review of the KPI process occurred.

**Results:**

Eighty-two cases were analysed. Domains with the highest percentage of achievement were: Indication 96%; Tube position confirmed 94% and Full AAGBI monitoring and Grade of view < 3 both 89%. The amount of missing data declined throughout the year. The results of the clinician survey showed that almost all respondents found the TVAA PHEA review process useful.

**Conclusion:**

The KPI process has demonstrated areas of good quality practice and led to improvements in equipment, processes and documentation and therefore patient care. We offer suggestions to other organisations considering implementing KPIs for PHEA.

## Key messages

### What is already known on this subject

Association of Anaesthetists of Great Britain and Ireland guidance from 2017 suggests the use of Key Performance Indicators to audit Pre-hospital Emergency Anaesthesia quality but does not specify how this should be done.

### What this study adds

Introduction of Key Performance Indicators (KPIs) improves performance in high risk procedures in Pre Hospital Care. This is the first article to describe how Key Performance Indicators for Pre Hospital Emergency Anaesthesia (PHEA) have been introduced in a service and it offers suggestions for other organisations wishing to do the same, whether they are pre hospital critical care teams or Emergency Departments.

## Background

Pre Hospital Emergency Anaesthesia (PHEA) is widely regarded as one of the highest risk interventions that Pre Hospital physicians perform. The process is complex and carries a significant risk of morbidity and mortality and it can be difficult to objectively measure and monitor performance of a Pre Hospital Care team delivering PHEA. Whilst there have been numerous standards and reviews covering in-hospital anaesthesia specific guidance on PHEA was first published by the AAGBI in 2009 [1], and this included a number of basic governance recommendations. As a result we already closely evaluated all PHEA performed by our service. The AAGBI updated this guidance in 2017 “AAGBI: Safer pre-hospital anaesthesia 2017” [2] and it now suggests developing Key Performance Indicators (KPIs) for PHEA.

The following items were suggested for use as KPIs in the AAGBI 2017 guidance [2]:

- **Structure/system**: routine use of a standard operating procedure and checklist for PHEA; all team. members familiar with the failed intubation plan; daily equipment checks performed; and full monitoring, including continuous waveform capnography available.
- **Process**: pre-oxygenation performed for 3 min; intubation performed by experienced airway provider; no decrease of more than 20% in systolic blood pressure; no decrease in SaO_2_ < 90%, or fall of > 10% from starting value; and no more than two attempts required for intubation.
- **Outcome**: position of tracheal tube maintained and confirmed using waveform capnography; adequate anaesthesia maintained during transfer; cardiovascular stability maintained; ventilation titrated to end-tidal CO_2_.

We are the first organization to develop KPIs based on the AAGBI 2017 guidance and publish the process and the results. In this paper, we present the methodology used to implement KPIs, how they have been adjusted in response to clinician feedback and how they have improved patient care in our organization. We also offer suggestions to other organisations wishing to develop a similar process.

## Method

### Study setting

Thames Valley Air Ambulance (TVAA) is an Air Ambulance organization working in the United Kingdom, operating in a mixed urban and rural area covering a population of approximately 2.2 million in an area of 5741 km^2^. During the study period TVAA operated both an EC135 helicopter available daily from 0700 to 1900 and a night service in partnership with Hampshire and Isle of Wight Air Ambulance covering the hours 1900–0200. TVAA also operated an Emergency Response Vehicle Car which was available from 0700 to 1900. Both platforms respond to taskings from the Emergency Operations Centre of South Central Ambulance Service (SCAS). TVAA respond to approximately 1300 taskings a year including trauma, medical cases and cardiac arrests. PHEA is provided by a physician and paramedic team, the intubator is either a physician or a paramedic operating under the direct supervision of a physician. At the time of the study our service employed 10 full time paramedics, 12 part time consultants and 9 part time registrars. During the study period TVAA was part of South Central Ambulance Service. Since the 1st October 2018 TVAA have been an independent healthcare provider and there have been changes to a number of organisational, logistical and clinical systems.

The study period was from 1 March 2017 to 28 February 2018. Under NHS guidance, this work is classed as service evaluation and Ethics Committee approval was not required.

### Study design

The domains suggested by the AAGBI guidelines were considered by an expert panel of NHS consultants in Anaesthesia and Emergency Medicine, all of whom work regularly with TVAA. The panel used the AAGBI document as well as expert opinion to devise the list of potential KPI domains, these are summarised in Table 1.

**Table 1:** Verdict of expert panel on items for inclusion as TVAA PHEA KPI

	Comments	Suggested inclusion on TVAA PHEA KPI
AAGBI Suggested KPI		
Use of SOP and checklist	Standard for all PHEA at TVAA	
All team familiar with failed intubation plans	Difficult to measure	
Daily equipment check performed	Data not retrievable from EPR	
Full monitoring including capnography	Zoll monitor links directly to EPR allowing easy review of these figures	Yes
Pre-oxygenation for 3 mins	Difficult to measure	
Intubation by experienced airway provider	All those providing PHEA at TVAA should be “experienced” as they will be either consultants or a minimum of ST4 in either Emergency Medicine or Anaesthesia	
No decrease of more than 20% in SBP	Zoll monitor links directly to EPR allowing easy review of these figures	Yes
No decrease in Sa0 _2_ below 90% or fall of > 10% below starting value	Zoll monitor links directly to EPR allowing easy review of these figures	Yes
No more than 2 attempts before success	The ‘RSI’ tab on the EPR includes this data, therefore easily measureable	Yes
Position of tube maintained and confirmed with capnography	Easily measured as Zoll monitor links directly to EPR	Yes
Adequate anaesthesia maintained	Difficult to define but TVAA SOPs suggest a continuous infusion of sedative via an infusion pump at a rate suitable for the patient	Yes
Cardiovascular stability maintained	Zoll monitor links directly to EPR allowing easy review of these figures	Yes
Ventilation titrated to ETCO _2_	Difficult to measure	
Other KPIs that were considered		
PHEA within 45 min of call	NICE standard for trauma. Also recommended in ref. 1	Yes
Indication for PHEA documented	The “RSI” tab on the EPR includes this data, therefore easily measureable Also recommended in ref. 1.	Yes
Grade of view	Cormack Lehane grade of view should be < 3. Consensus opinion by expert group to detect patterns potentially due to poor intubating technique.	Yes

The expert panel recommended ten domains to be included in the KPI:

1. Full monitoring (ECG, respiratory rate, oxygen saturations, systolic and diastolic BP, ETCO_2_)
2. No decrease of > 20% in systolic BP at induction
3. No decease in SaO_2_ below 90% or fall of 10% below starting value at induction
4. No more than 2 attempts before successful tracheal intubation
5. Position of tube maintained and confirmed with capnography
6. Adequate anaesthesia maintained throughout transfer
7. Cardiovascular stability maintained throughout transfer
8. PHEA within 45 min of call. This is a UK national standard set by NICE for trauma cases [3]
9. Indication for PHEA documented
10. Grade of laryngeal view should be < 3

### Data collection

**Fig. 1:**
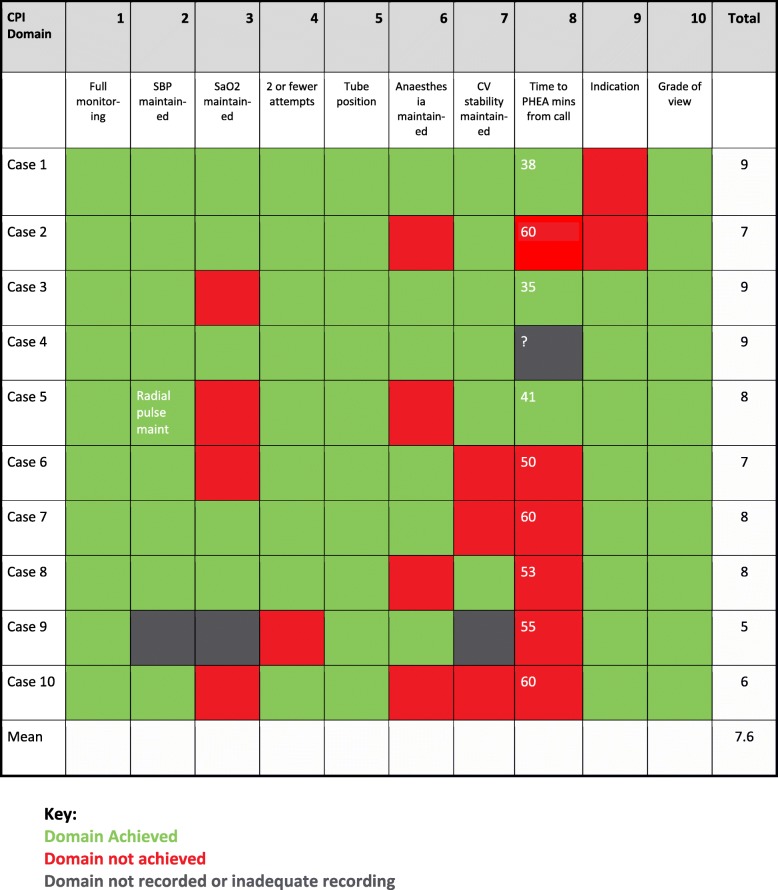
TVAA PHEA KPI matrix for a sample month

The Zoll X series monitors (Zoll, Runcorn, UK) used by TVAA uploads the patient’s vital signs directly into the SCAS Electronic Patient Record (EPR) (Panasonic, Loughborough UK). The EPR contains specific fields for details of any PHEA event known as the ‘RSI tab’. Data from these fields can be extracted by the SCAS business intelligence team directly from the cloud-based EPR into an Excel® (Microsoft, Redmond, US) spreadsheet. At the end of each month data from all PHEA conducted that month are analysed, each case is assessed according to the 10 domains and each domain is given a score of one if the domain has been achieved or zero if the domain was not achieved or if the data was missing. These scores are then added together to produce a total for each case which can be between zero (no domains were achieved) and ten (all domains achieved). This process is repeated for each case and the data entered into a matrix, with a green box where a domain was achieved, a red box if a domain was not achieved and a grey box if data were missing. At the end of each month an overall mean score of all PHEA is calculated and this value plotted on a run chart. An example of the matrix for a typical month is at Fig. 1.

### Primary and secondary endpoints

Each PHEA case is discussed during the monthly multidisciplinary clinical governance and case review meeting and any cases with low scores or any trends are subject to extra scrutiny. The results of the case review meeting, the graphical matrix and the run chart of monthly mean PHEA KPI scores are then circulated to all TVAA clinicians as part of the minutes of the review meeting.

### Process evaluation

Following a year of PHEA KPIs all TVAA clinicians were surveyed to determine their views on the process and utility of the PHEA KPIs, clinicians were invited to take part in a web based survey using Google Forms, the invitation was sent by email and via the TVAA WhatsApp chat group and non -responders were reminded by email and WhatsApp after two weeks. The survey asked questions on the overall monthly case review process as well as specific questions regarding the PHEA KPI process.

The collated results of a year of PHEA KPIs were presented at a governance meeting and an open forum was held where suggestions for refinement of the process were invited.

## Results

The domains suggested by the AAGBI guidelines were considered by an expert panel of NHS consultants in Anaesthesia and Emergency Medicine, all of whom work regularly with TVAA. The panel used the AAGBI document as well as expert opinion to devise the list of potential KPI domains, these are summarised in Table 1.

**Fig. 2:**
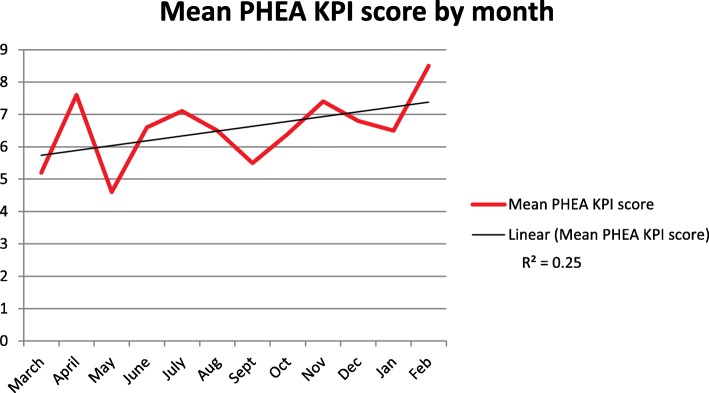
Run chart of mean score of first year of PHEA KPI results

At the end of the first year of using PHEA KPIs at TVAA a total of 83 PHEA cases had been conducted, one case was excluded due to complete lack of data, the results of 82 cases were analysed. 52 (63%) of the cases were trauma, 11 (13%) were medical and 19 (23%) were cardiac arrest patients with return of spontaneous circulation. Figure 2 shows the run chart of the mean score for each month.

The regression line was calculated using Microsoft Excel, the r squared value was 0.25.

Collating the results for the entire year, the domains with the highest percentage of achievement were:

- *Indication* 96%;
- *Tube position confirmed* − 94%
- *Full AAGBI monitoring - *89%
- *Grade of view < 3* both - 89%.

Domains with the lowest level of achievement were

- *PHEA within 45 min of call - 62% not achieved*
- *Anaesthesia maintained throughout transfer - 38% not achieved.*

The domains with the highest proportion of missing data were

**Fig. 3:**
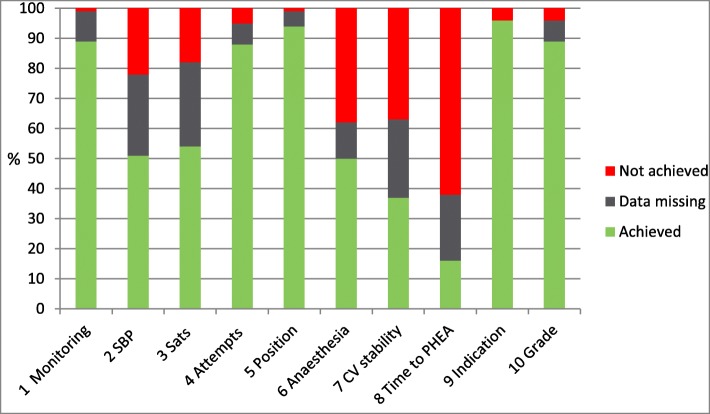
Percentage achievement of KPI by domain over one year

- *No decease in SaO2 below 90% or fall of 10% below starting value at induction - 28%*
- *No decrease of > 20% in systolic BP at induction - 27%*
- *Cardiovascular stability maintained throughout transfer - 27%* (Fig. 3)

**Fig. 4:**
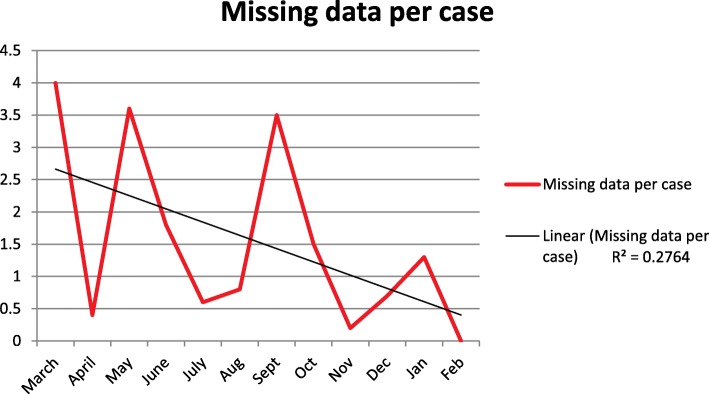
Missing data per case

The amount of missing data declined throughout the year as clinicians became more aware of the data gathering requirements of the KPIs and better at using the EPR. Figure 4 shows the number of missing data fields per case by month.

**Fig. 5:**
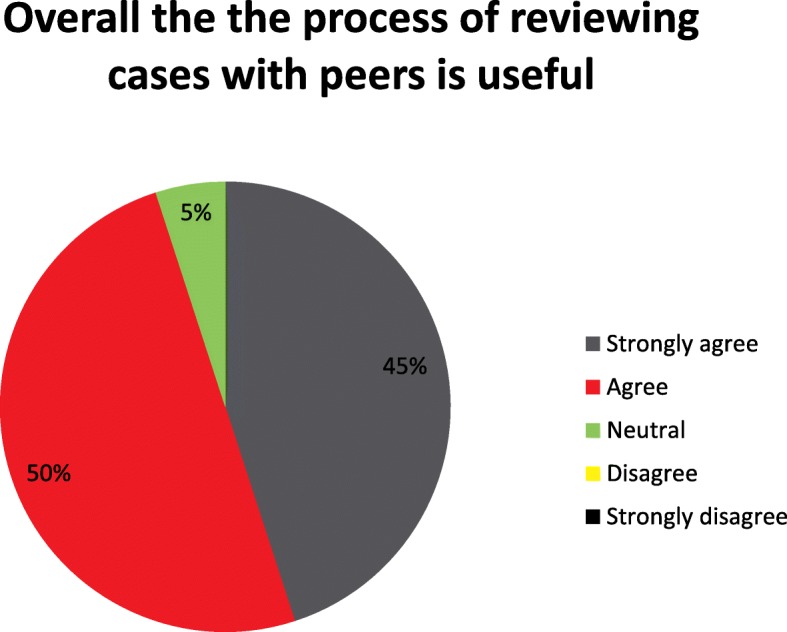
Survey question – overall the process of reviewing cases with peers is helpful

**Fig. 6:**
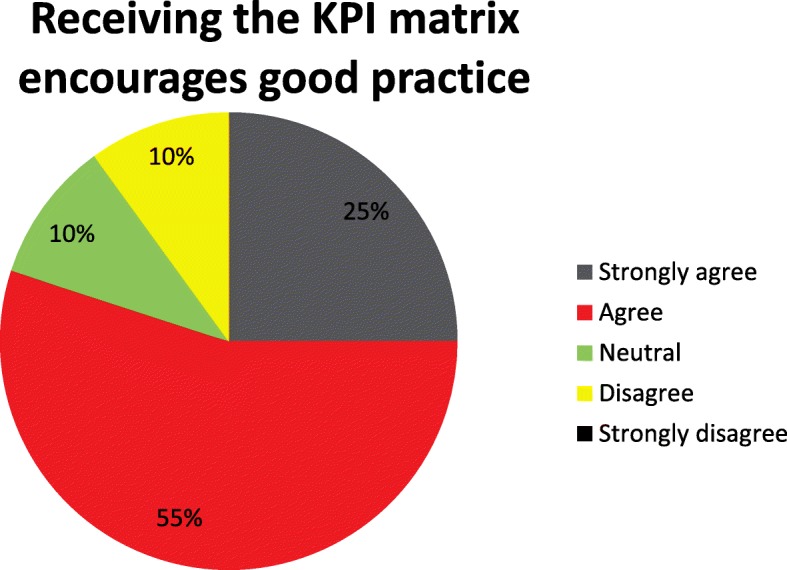
Survey question - KPIs encourage good practice

20 clinicians (4 of 10 paramedics, 7 of 12 consultants and 9 of 9 registrars) responded to the survey. The results showed that almost all respondents found the TVAA PHEA review process useful (Fig. 5), 9/20 respondents strongly agreed and 10/20 respondents agreed, one was neutral and no respondents disagreed. 80% felt that the KPI matrix encouraged good practice, 5/20 respondents strongly agreed and 11/20 respondents agreed, two were neutral and two respondents disagreed, no one strongly disagreed (Fig. 6).

Positive free text comments on the KPI process included “clear graphics allow easy interpretation”, “KPIs maintain practice quality” and “A good process for individual and system learning”. The survey asked “If KPIs have changed your practice please describe how?” Replies focussed on the discussions about maintenance of anaesthesia that happened at the review meetings and were documented in the minutes, with clinicians commenting that practice had changed as a result of this. Other comments included the question “Does achieving KPIs reflect good practice?” and “Targets may not be achieved for valid reasons”.

## Discussion

TVAA are the first air ambulance organisation to introduce KPIs for PHEA based on the AAGBI 2017 recommendations. As an organization we feel that in order to improve patient care in the pre-hospital setting, guidance and latest evidence should be adhered to and we regard the KPI process as a way of gently guiding clinicians in the right direction to improve clinical practice. The Santana group identified nine dimensions that KPIs should meet [4], these are:

- Targets important improvements
- Precisely defined
- Reliable
- Valid
- Can be implemented with risk adjustment
- Can be implemented with reasonable cost
- Data collection effort is low
- Results easily interpreted
- Globally is a good indicator (overall evaluation)

In addition, KPIs should be evidenced based wherever possible. Collating and disseminating the KPIs is in keeping with the behavioural science of ‘nudge theory’, developed by Richard Thaler [5]. As the project was implemented, we found we needed to educate clinicians on the best method of recording data on the EPR and produced a flowchart so that data items were reliably recorded in the same place for each case. We also ensured that clinicians were aware of the relevant parts of our guidelines and of how the data were used to create the matrix by including reminders in the monthly case review minutes, as well as going over the relevant sections during the meetings. Organisational and financial considerations included increasing the time allotted for the case review process, this included an increase in the time spent gathering the data from the EPR prior to the meeting, as well as longer review meetings in which to discuss the cases adequately. As with any new quality improvement measure we did encounter resistance to change, however this was minimized by ensuring that the process was seen as open and constructive, encouraging all clinicians to contribute in cases where they had been involved and constantly seeking feedback on the process.

Domains with the highest levels of completion were those where it was easy for the clinician to record the data, *Indication, Tube position confirmed* and *Grade of view* are all tabs with drop down menus on the EPR. Domains with the lowest level of achievement were *PHEA within 45 min of call* and *Anaesthesia maintained*. The target of PHEA within 45 min of call attracted a lot of comment and discussion throughout the year. This target was set by the UK National Institute for Health and Care Excellence (NICE) [3] as an aspirational target to challenge the performance of pre hospital critical care providers. Some clinicians within the service felt that it was an unfair measure as it is down to whole system performance which involves four phases; case identification within the EOC; TVAA dispatch and mobilization; travel to scene time and on scene time assessing and preparing the patient. Some clinicians have suggested that just the on scene time is used as the performance indicator; however the importance of having an overall measure of system performance was recognized, especially as this domain now forms part of the NICE Performance Indicators for Major Trauma [6] (although we also applied this 45 min target to medical and cardiac arrest cases with return of spontaneous circulation) and clinicians were reminded that this was not criticising individual doctors or paramedics, but rather gave an overview of the multiple system elements required to provide PHEA. Analysis of this data has also led to the launch of a project looking specifically at improving call to PHEA times. The difficulty of achieving this target has been identified by other Air Ambulance services [7].

The maintenance of anaesthesia domain also attracted a lot of discussion at all levels and this debate led to changes in the way that anaesthesia is maintained after induction and the introduction of new anaesthetic pumps. We deliberately set a high standard to achieve a domain, and initially any maintenance of anaesthesia that didn’t exactly meet the definition in the relevant guideline was coded as a domain not achieved. As the year progressed it became evident that the wording of the guideline did not reflect the variety and complexity of the cases that we see and as a result the guideline was updated and amended.

The domains with the highest proportion of missing data were *No decrease in SaO*_*2*_ *below 90% or fall of 10% below starting value*; *No decrease of > 20% in systolic BP at induction* and *Cardiovascular stability maintained throughout transfer*. Achievement of all these domains relies on the transfer of data from the Zoll monitor to the EPR via a wireless link. There have been a number of cases where this link has failed or the monitor has been inadvertently shut down prior to data transfer leading to missing data. Further analysis of domain 3 *No decease in SaO*_*2*_ *below 90% or fall of 10% below starting value* also led us to recognize a fault with the oxygen saturation probe on the monitors which has led us to source replacements. Review of a number of cases where cardiovascular stability was not maintained led to an amendment to the KPI process to recognize those cases where cardiovascular instability had been present during the entire case, including before anaesthesia, by annotating the matrix with a comment such as “unstable throughout” or “radial pulse maintained” to add context in cases where instability was felt to be due to pathology rather than the process of anaesthesia.

Following the presentation and discussion of the collated results of a year of PHEA KPIs domains, the list was finalized with a specified time added to domain 2, use of capnography (domain 5) altered to include the ETCO_2_ being within target range on arrival at hospital and in domain 7 cardiovascular instability was defined more clearly. The final domains are shown with changes and additions from the original highlighted in italics

1. Full monitoring (pulse rate, resp. rate, O_2_ sats, systolic and diastolic BP, ETCO_2_)
2. No decrease of > 20% in SBP at induction *or in the following 3 min*
3. No decrease in SaO_2_ below 90% or fall of 10% below starting value at induction
4. No more than 2 attempts before success
5. *ETCO*_*2*_ *used and within target range (3.5–5.0 kPa) on arrival at hospital*
6. Adequate anaesthesia maintained throughout transfer
7. Cardiovascular stability maintained throughout transfer. (*Instability is defined as a drop of > 20% in SBP or DBP*)
8. PHEA within 45 min of call
9. Indication for PHEA documented
10. Grade of View should be < 3

The KPI process drove noticeable improvements in the standard of completion of the EPR and this trend is shown in Fig. 4. We did not find any published results of other services implementing KPIs for PHEA; however KPIs are increasingly used, particularly for high risk interventions and as a valuable tool for improving patient care.

### Implementation in TVAA and other services

We encountered some specific challenges in implementing the KPI process, it took time to gain acceptance from clinicians and we produced frequent reminders that:

- The process was not a pass/fail exercise
- There would always be some difficult cases where the achievement of a high score would be impossible.
- Good clinical performance was noted irrespective of the numerical score assigned to a case.
- In addition to this we encouraged all clinicians to attend the monthly case review meetings, especially if one of their cases was being discussed, so they could comment on and explain findings and to encourage an open and collaborative atmosphere. We offer the following suggestions to other organisations considering implementing KPIs for PHEA:
- Ensure all clinicians feel involved in the creation and development of the programme, seek their contribution at all stages and invite input on a frequent basis.
- Tailor the process to your own organization and the environment you operate in.
- Adapt the process in response to feedback and the identification of trends.
- Maintain an open and non-judgmental atmosphere at the meetings, especially when the clinicians involved cannot be present at the monthly case review. When we identified particularly challenging cases, we would contact the clinicians involved in advance of the meeting to seek clarity and avoid misinterpretation.

The 2017 AAGBI guidelines are an important step in improving the quality and safety of PHEA, the development of service specific KPIs is a logical step in improving patient care and could be implemented in the Emergency Department Resuscitation Room as well as in the Pre Hospital environment.

### Limitations

This is a single centre study and we specifically targeted the domains to our clinician skill mix and case mix, other services wishing to implement KPIs should consider their local operating environment when selecting domains. The use of a binary system for the outcomes of domains 2, 3 and 7 does not reflect the complexity of a critically ill patient and we adapted the process to include comments where the patient had been cardiovascularly unstable throughout the case. The number of cases does not allow for detailed statistical analysis, but the trends are clear for the data we have analysed.

## Conclusion

The use of KPIs for PHEA has focused attention on the conduct of PHEA at TVAA and driven improvements in both the practice and record keeping of PHEA. It has also added objectivity to an otherwise subjective review process.

We identified trends of poorly performing areas, leading to equipment upgrades, clinician education, further studies of system performance and improvements in completion of the EPR. Feedback on the process and on the presentation of results was positive. Our suggestions for other organisations wishing to implement a similar process at their institution include ensuring clinicians feel involved throughout, adapting the process according to your environment and frequent reviews of the process with an open and non-judgemental atmosphere at the review meetings.
